# Supplementary material for: Flow analysis on microcasting with degassed polydimethylsiloxane micro-channels for cell patterning with cross-linked albumin
Source: PLoS One. 2020 May 20;15(5):e0232518. doi: 10.1371/journal.pone.0232518 (PMC7239381; doi:10.1371/journal.pone.0232518)
Supplement: S1 Text — (DOCX) [file pone.0232518.s001.docx]

**Supporting information for the following paper:**

**Flow analysis on microcasting with degassed polydimethylsiloxane micro-channels for cell patterning with cross-linked albumin**

Yigang Shen^1,2¶^, Nobuyuki Tanaka^1¶^, Hironori Yamazoe^3^, Shunsuke Furutani^3, 4^, Hidenori Nagai^3, 4^, Takayuki Kawai^1^, and Yo Tanaka^1,2*^

^1^RIKEN Center for Biosystems Dynamics Research, Osaka, Japan

^2^Graduate School of Frontier Biosciences, Osaka University, Suita, Osaka, Japan

^3^Biomedical Research Institute, National Institute of Advanced Industrial Science and Technology (AIST), Osaka, Japan

^4^Advanced Photonics and Biosensing Open Innovation Laboratory (PhotoBIO-OIL), AIST, Osaka, Japan

^¶^These authors contributed equally to this work

* yo.tanaka@riken.jp

**S1 Text. A method for determining the area of cell attachment.**

**Image processing**

ImageJ was used as the platform to calculate the cell region, cell blocking region and non-specific cell region. The process used three functions of ImageJ: convert to grayscale, regulate the threshold value (that is, find the edges) and analyze particles (Fig S1-1). All the original images are concluded in supporting Information S1 File.

**Conditions of the cell grown in the area of the cross-linked pattern**

The results of image processing indicated that the non-specific cell adhesion region was influenced by the geometrical dimensions of the pattern (a wider channel had a higher area ratio than a narrow channel) (Fig S1-2), and this region was thought to be caused by defects formed during the casting process.
