# Supplementary material for: Flow analysis on microcasting with degassed polydimethylsiloxane micro-channels for cell patterning with cross-linked albumin
Source: PLoS One. 2020 May 20;15(5):e0232518. doi: 10.1371/journal.pone.0232518 (PMC7239381; doi:10.1371/journal.pone.0232518)
Supplement: S2 Text — (DOCX) [file pone.0232518.s002.docx]

**Supporting information for the following paper:**

**Flow analysis on microcasting with degassed polydimethylsiloxane micro-channels for cell patterning with cross-linked albumin**

Yigang Shen^1,2¶^, Nobuyuki Tanaka^1¶^, Hironori Yamazoe^3^, Shunsuke Furutani^3, 4^, Hidenori Nagai^3, 4^, Takayuki Kawai^1^, and Yo Tanaka^1,2*^

^1^RIKEN Center for Biosystems Dynamics Research, Osaka, Japan

^2^Graduate School of Frontier Biosciences, Osaka University, Suita, Osaka, Japan

^3^Biomedical Research Institute, National Institute of Advanced Industrial Science and Technology (AIST), Osaka, Japan

^4^Advanced Photonics and Biosensing Open Innovation Laboratory (PhotoBIO-OIL), AIST, Osaka, Japan

^¶^These authors contributed equally to this work

* yo.tanaka@riken.jp

**S2 Text. Numerical computation of liquid flow in micro-casting with a degassed PDMS mold.**

**Simulation**

Hagen–Poiseuille-like ﬂuid ﬂow is supposed, and then the flow in a rectangular cross-sectional micro-channel can be described as the following ordinary differential equations [27].

$F_{air}\text{=}D\left.\frac{\partial C}{\partial x}\right|_{x=t}\approx K_{1}*2D\left(\frac{C_{1}-C_{0}}{L_{PDMS}}\right)\text{exp}\left(\frac{\text{-}\pi^{2}D\left(t+t_{idle}\right)}{\text{4L}_{\text{PDMS}}^{2}}\right)$ (S1)

$R_{f}=K_{2}*\frac{12\eta L\left(W+H\right)^{2}}{\left(WH\right)^{3}\left(1+\frac{5H}{6W}\right)}$ (S2)

$Q=\frac{\Delta P}{R_{f}}$ (S3)

Ideal gas behavior is assumed, and the equation of gaseous state in the micro-channel can be written as follows.

$\Delta P=P_{atm}-\frac{n_{channel}RT}{V_{L}}+P_{c}$ (S4)

The air volume and surface area exposed in the micro-channel can be shown through the following geometrical relationships.

$V_{air}=WHL-\int_{0}^{t} Qdt$ (S5)

$A_{air}=LW+2LH+WH-\frac{W+2H}{WH}\int_{0}^{t} Qdt$ (S6)

Based on these relationships, the equation S1 can be introduced into the ordinary differential equations as follows.

$\frac{dA_{air}}{dt}=-\frac{W+2H}{WH}Q$ (S7)

$\frac{dV_{air}}{dt}=-Q$ (S8)

$\frac{dn_{c}}{dt}=-K_{1}2D_{PDMS}\left(\frac{C_{atm}-C_{PDMS}}{L_{PDMS}}\right)\text{exp}\left(\frac{\text{-}\pi^{2}D_{PDMS}\left(t+t_{idle}\right)}{\text{4L}_{\text{PDMS}}^{2}}\right)A_{air}$ (S9)

$\frac{dQ}{dt}=-K_{2}\left(\frac{\left(WH\right)^{3}\left(1+\frac{5H}{6W}\right)}{12\eta L\left(W+H\right)^{2}}\right)RT\left(\left(\frac{1}{V_{free}}\right)\frac{dn_{c}}{dt}-\left(\frac{n}{V_{free}^{2}}\right)\frac{dV_{free}}{dt}\right)$ (S10)

And the initial conditions are set as follows.

$A_{air0}=2L(W+H)+A_{end}$ (S11)

$V_{air0}=WHL$ (S12)

$n_{c0}=\frac{P_{atm}WHL}{RT}$ (S13)

$Q_{0}=0$ (S14)

The parameters in these equations were chosen to correspond to the experimental conditions or obtained from the literature [37]:

In order to solve the ordinary differential equations, firstly, two empirical factors K_1_ and K_2_ were determined by fitting experimental data. Here, two steps are offered to find the best-fit empirical factors: firstly, using the method of least squares to find the range of K_1_ and K_2_ ; Secondly, adjusting the empirical factors manually based on the experimental data. Then, the two determined factors was used for obtaining the solutions of the ordinary differential equations by numerical computation. The software was coded in the programing language Python 3.5.1 with an open source platform (Anaconda 4.0.0) (Continuum Analytics, Austin, TX) including a scientific computing library (SciPy 1.2.1), a numerical computing library (NumPy 1.10.4), a non-linear least-squares minimization and curve-fitting library (Lmfit 0.9.13). The main code and data are included in supporting Information S2 File.
