# Supplementary figures and images for: Flow analysis on microcasting with degassed polydimethylsiloxane micro-channels for cell patterning with cross-linked albumin
Source: PLoS One. 2020 May 20;15(5):e0232518. doi: 10.1371/journal.pone.0232518 (PMC7239381; doi:10.1371/journal.pone.0232518)

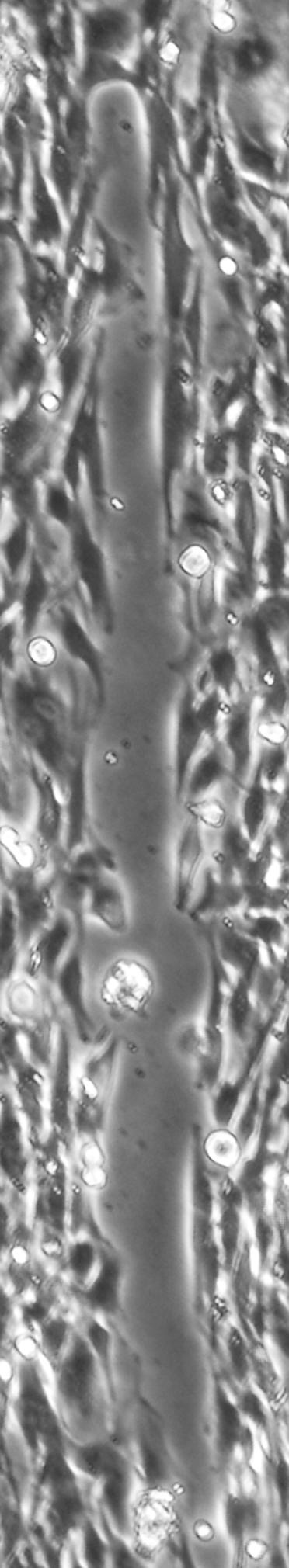

Supplement: S1 File — (ZIP) [file pone.0232518.s003.zip › 1 day/0.jpg]

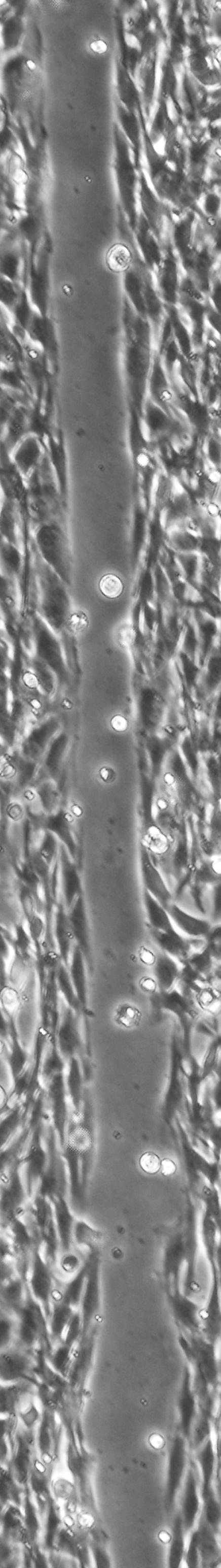

Supplement: S1 File — (ZIP) [file pone.0232518.s003.zip › 1 day/1.jpg]

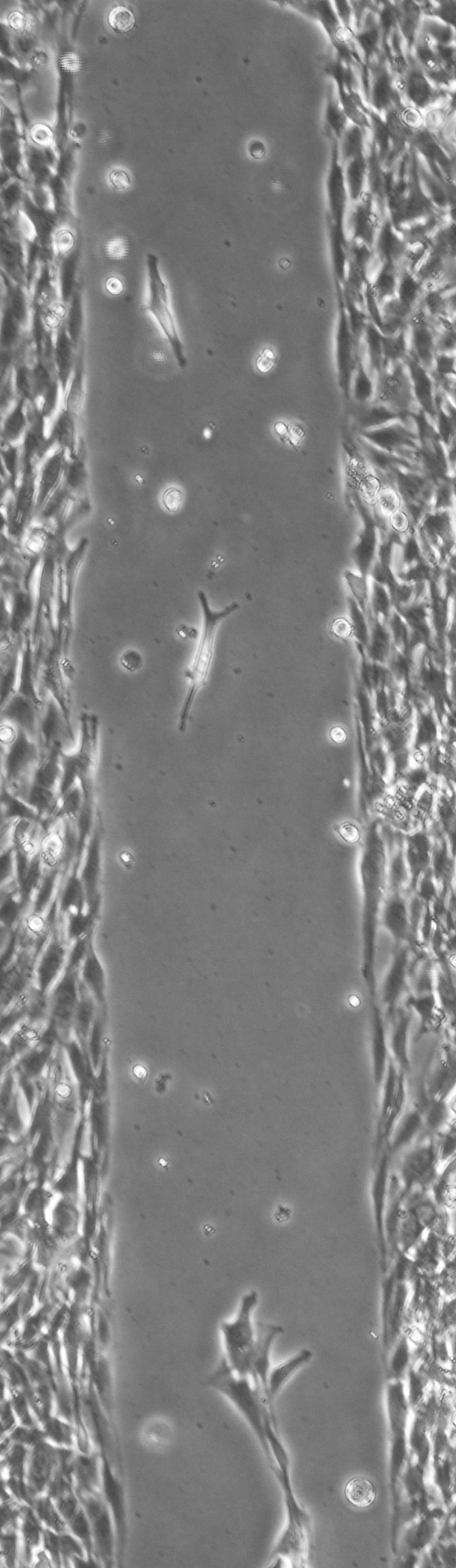

Supplement: S1 File — (ZIP) [file pone.0232518.s003.zip › 1 day/2.jpg]

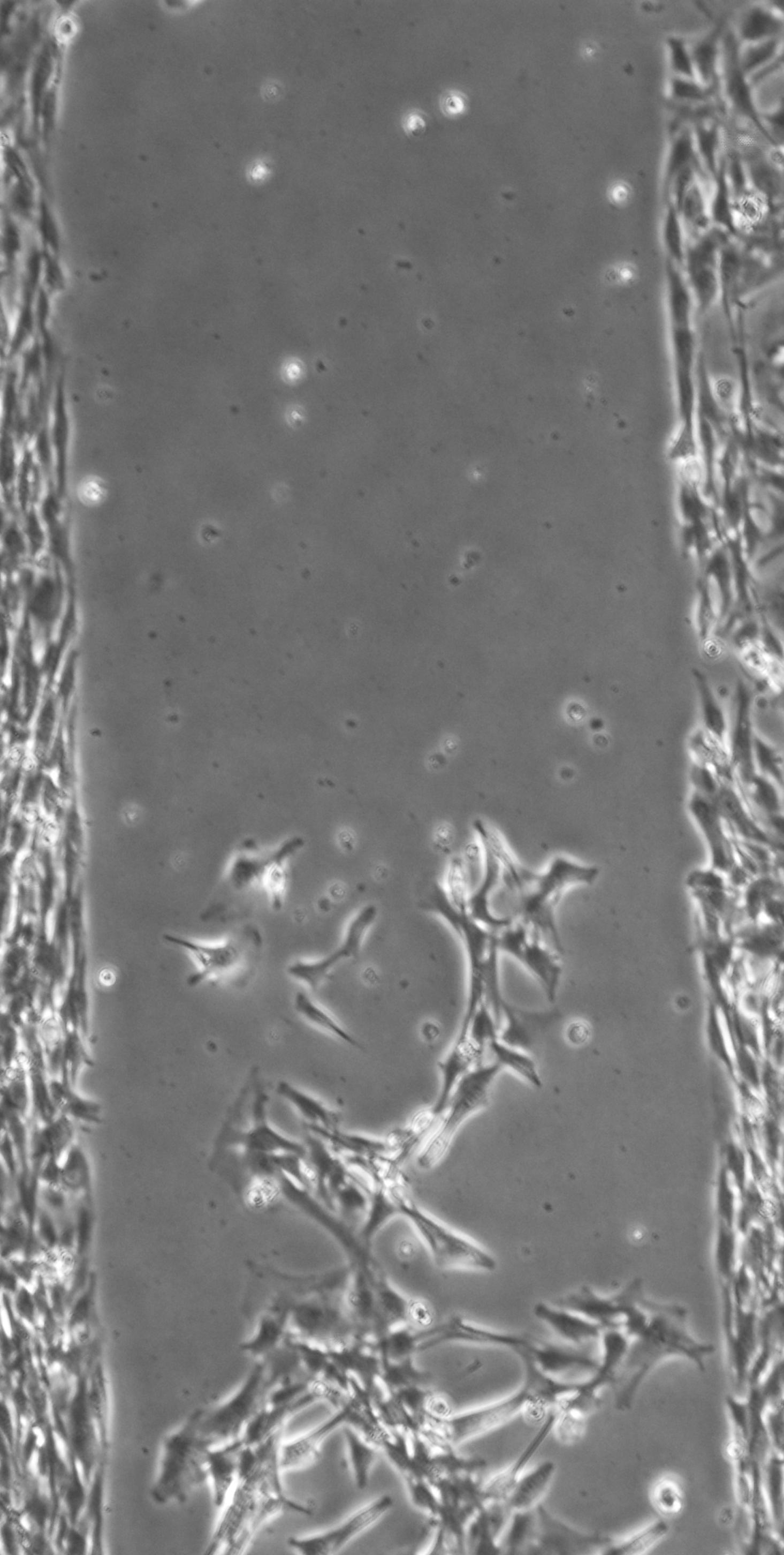

Supplement: S1 File — (ZIP) [file pone.0232518.s003.zip › 1 day/3.jpg]

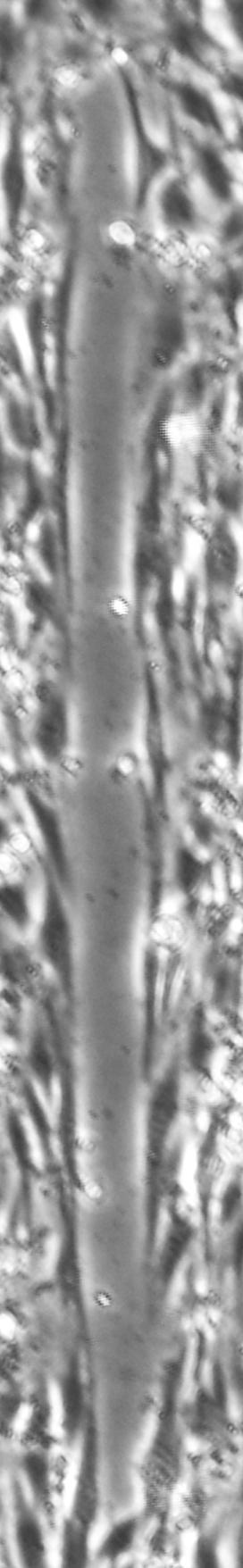

Supplement: S1 File — (ZIP) [file pone.0232518.s003.zip › 2 day/0.jpg]

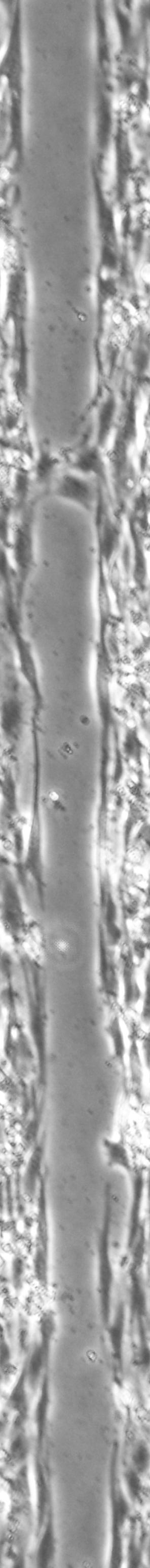

Supplement: S1 File — (ZIP) [file pone.0232518.s003.zip › 2 day/1.jpg]

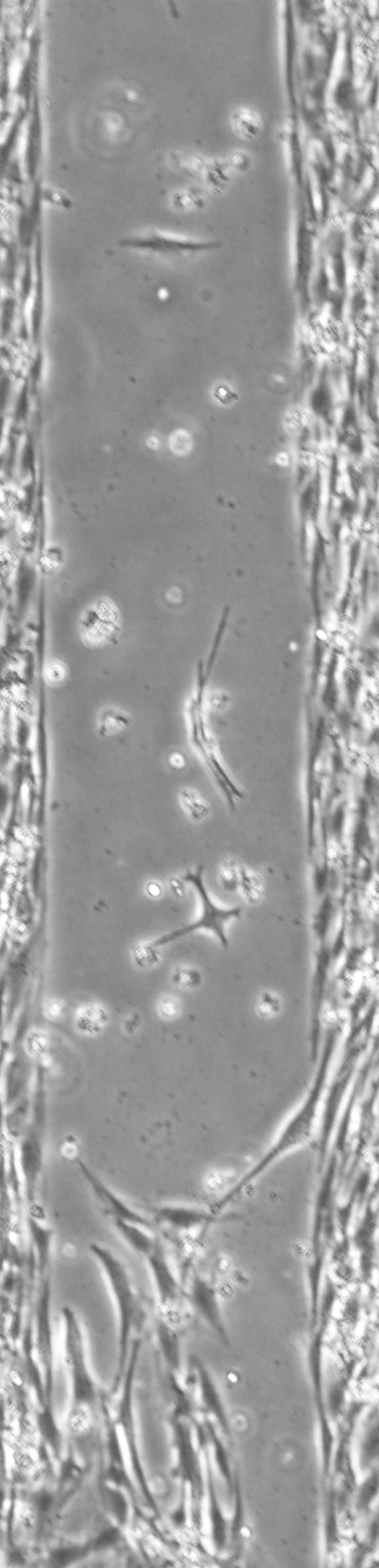

Supplement: S1 File — (ZIP) [file pone.0232518.s003.zip › 2 day/2.jpg]

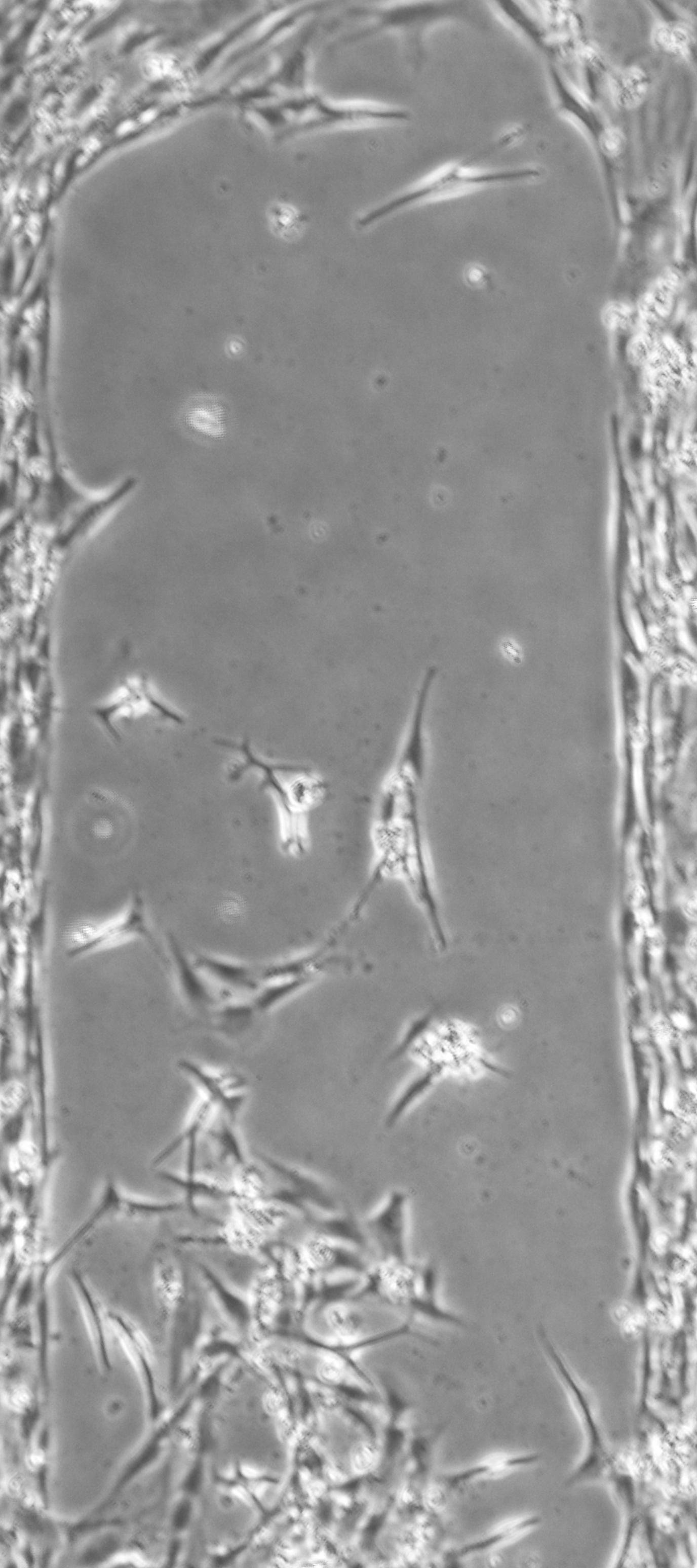

Supplement: S1 File — (ZIP) [file pone.0232518.s003.zip › 2 day/3.jpg]

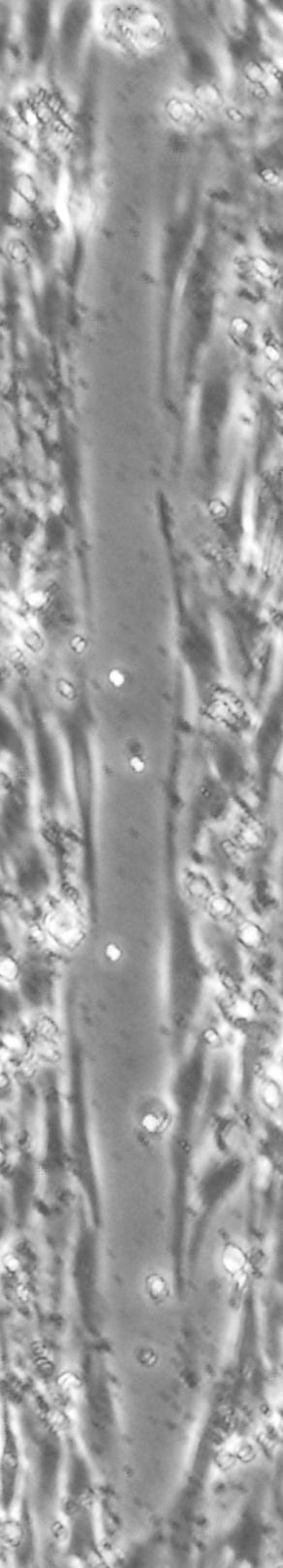

Supplement: S1 File — (ZIP) [file pone.0232518.s003.zip › 3 day/0.jpg]

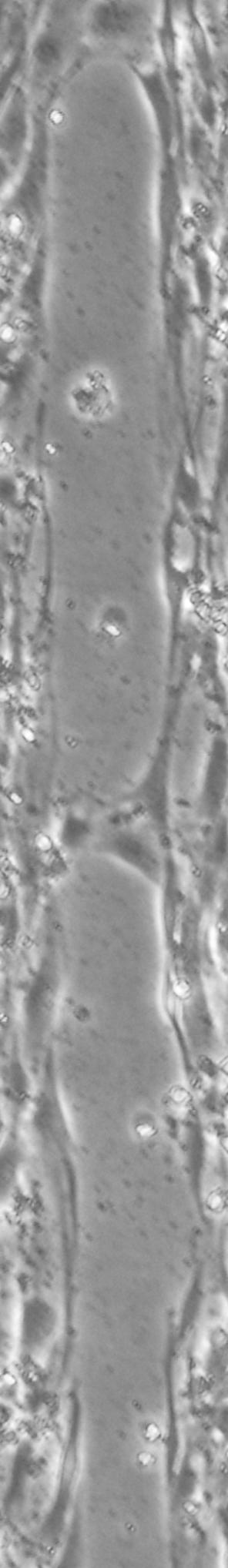

Supplement: S1 File — (ZIP) [file pone.0232518.s003.zip › 3 day/1.jpg]

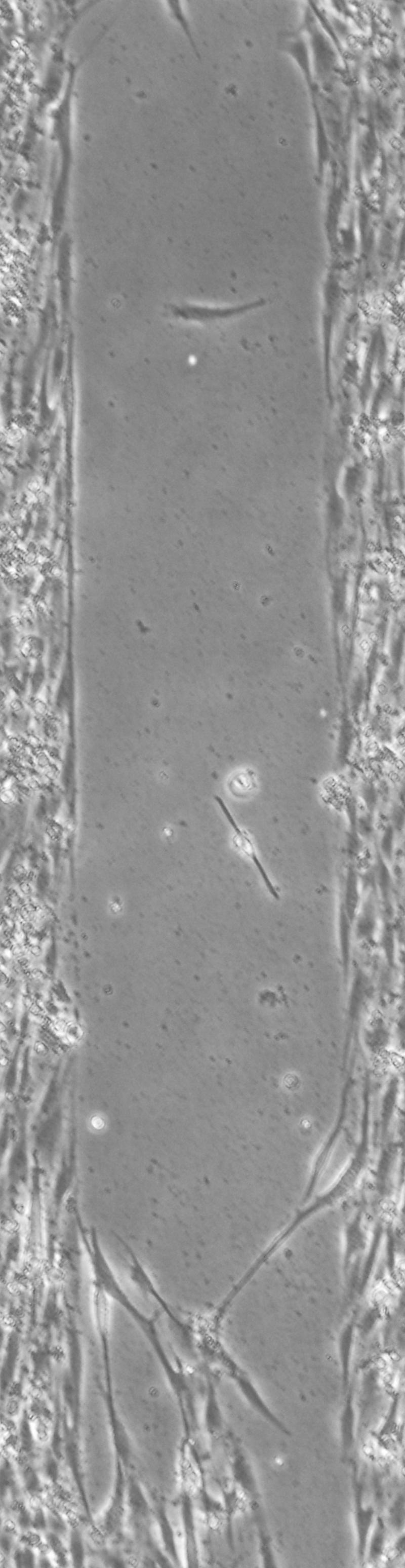

Supplement: S1 File — (ZIP) [file pone.0232518.s003.zip › 3 day/2.jpg]

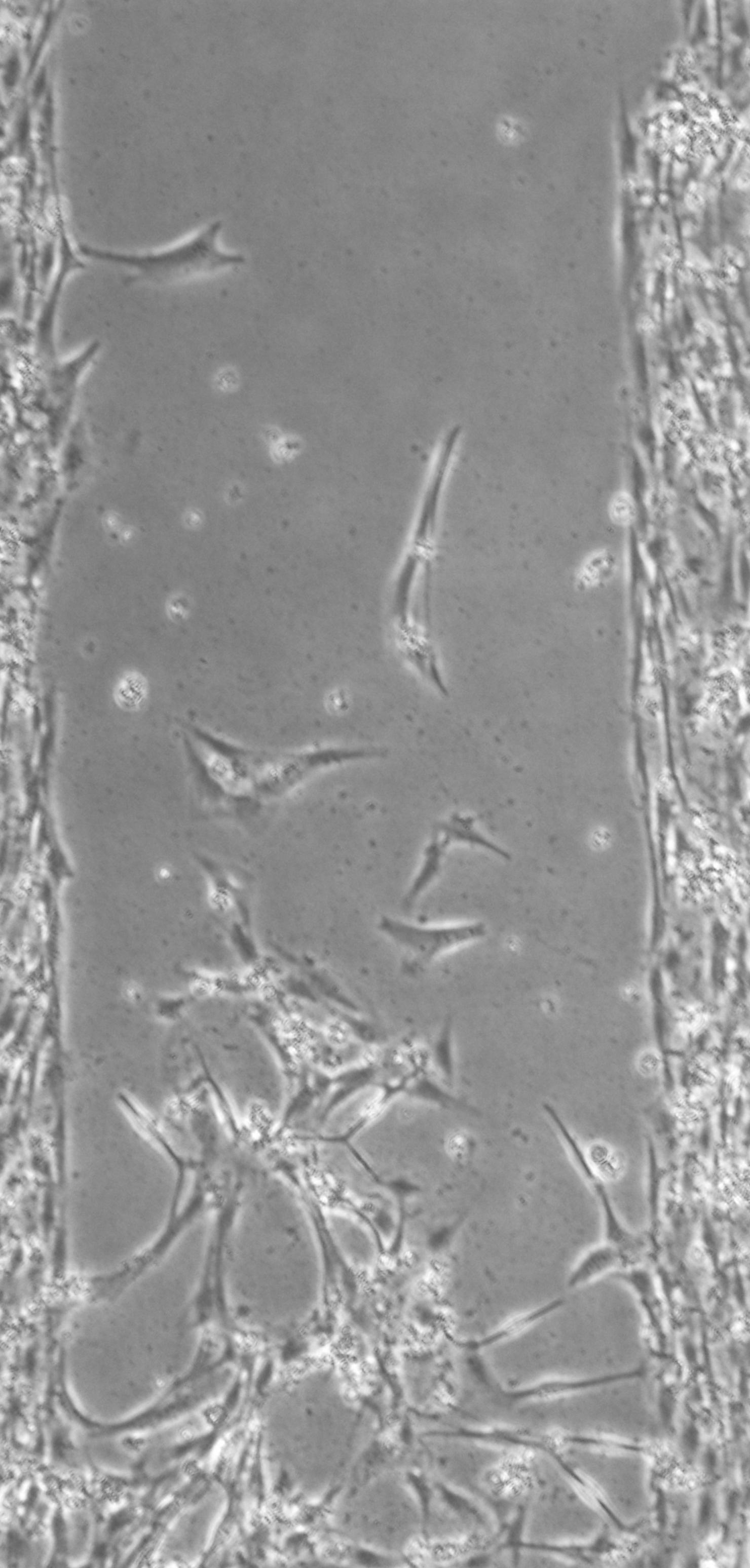

Supplement: S1 File — (ZIP) [file pone.0232518.s003.zip › 3 day/3.jpg]

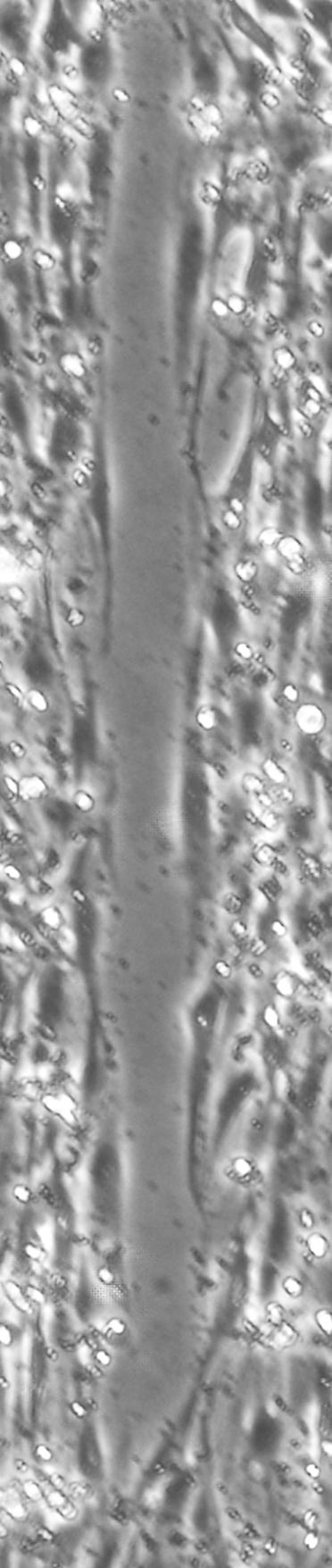

Supplement: S1 File — (ZIP) [file pone.0232518.s003.zip › 4 day/0.jpg]

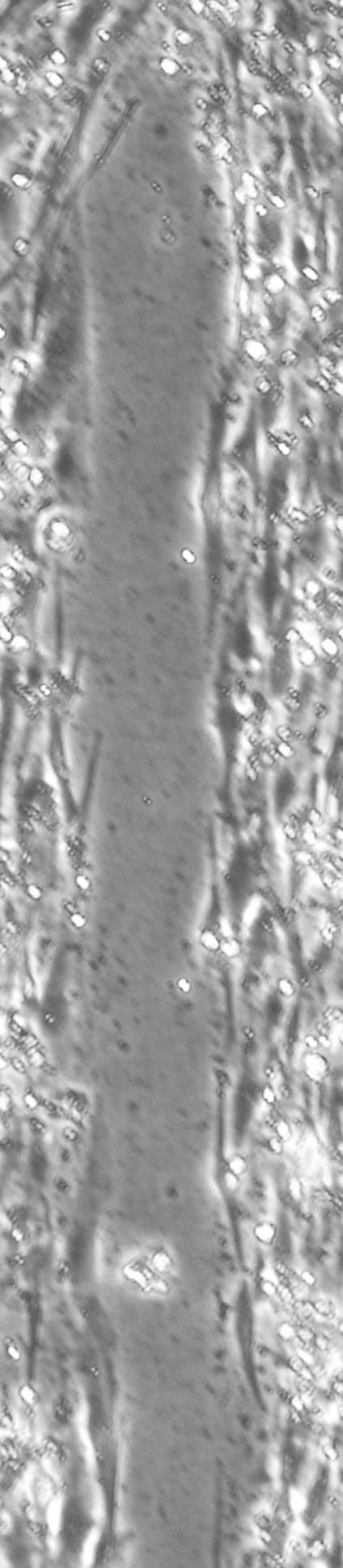

Supplement: S1 File — (ZIP) [file pone.0232518.s003.zip › 4 day/1.jpg]

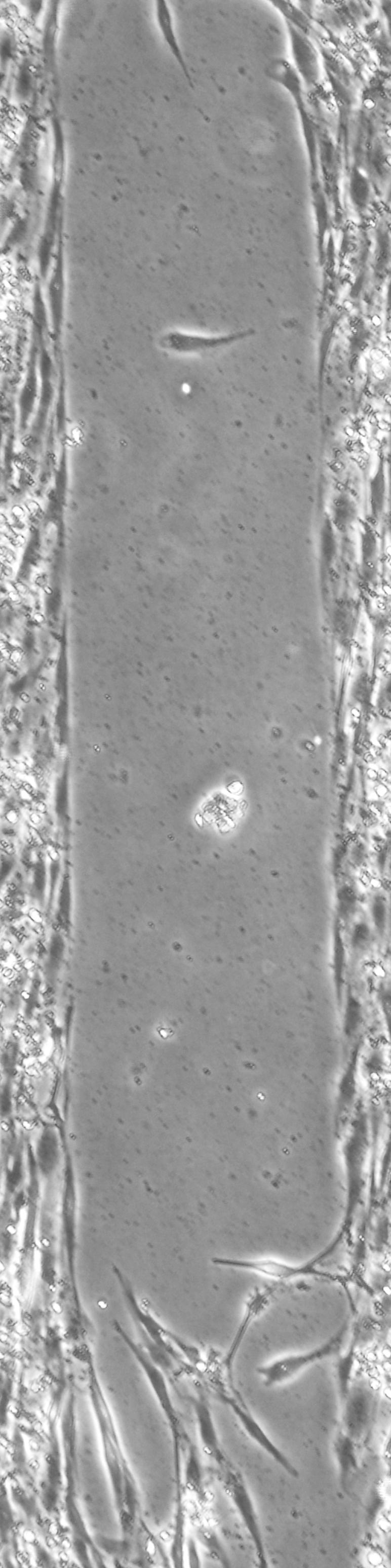

Supplement: S1 File — (ZIP) [file pone.0232518.s003.zip › 4 day/2.jpg]

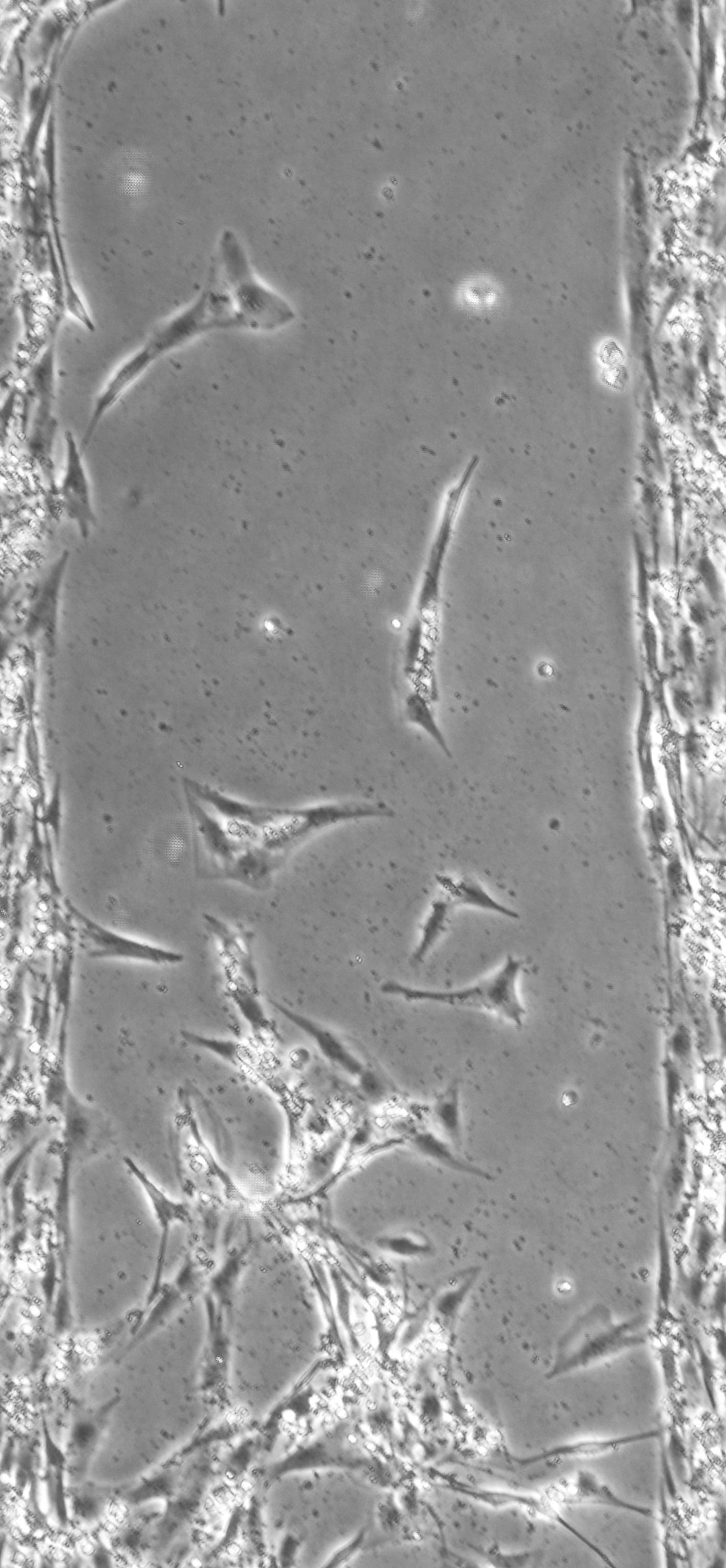

Supplement: S1 File — (ZIP) [file pone.0232518.s003.zip › 4 day/3.jpg]

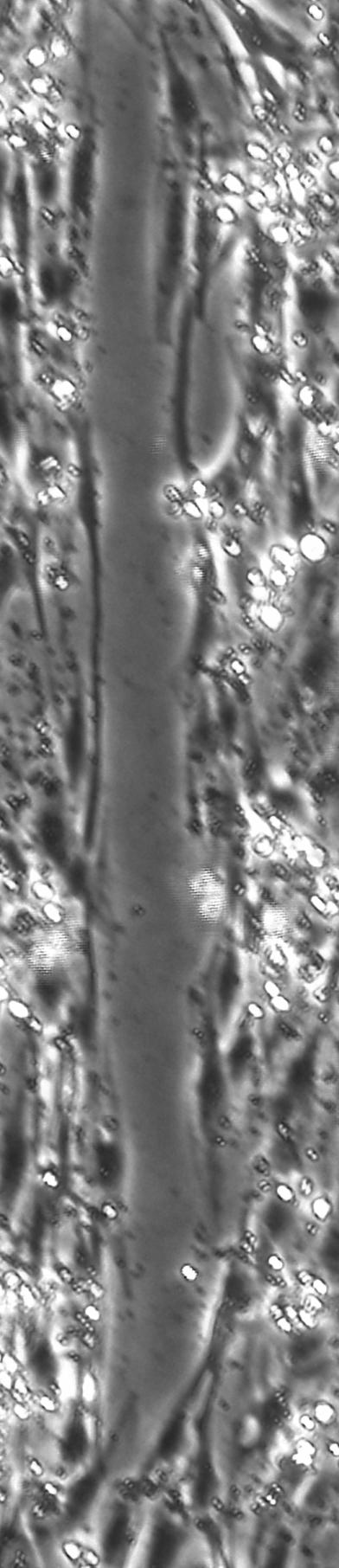

Supplement: S1 File — (ZIP) [file pone.0232518.s003.zip › 5 day/0.jpg]

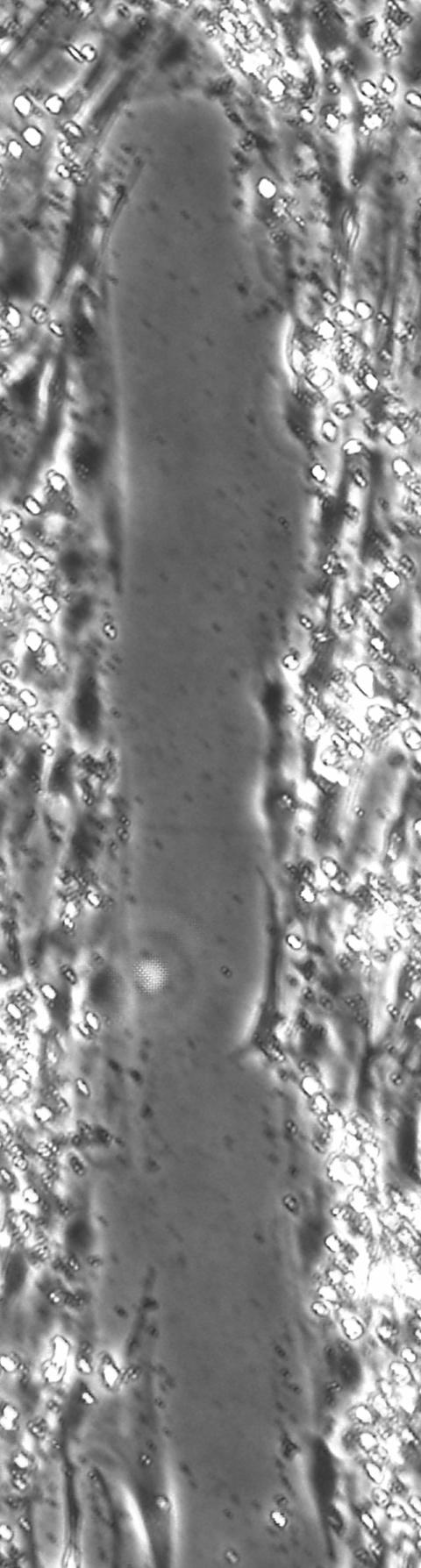

Supplement: S1 File — (ZIP) [file pone.0232518.s003.zip › 5 day/1.jpg]

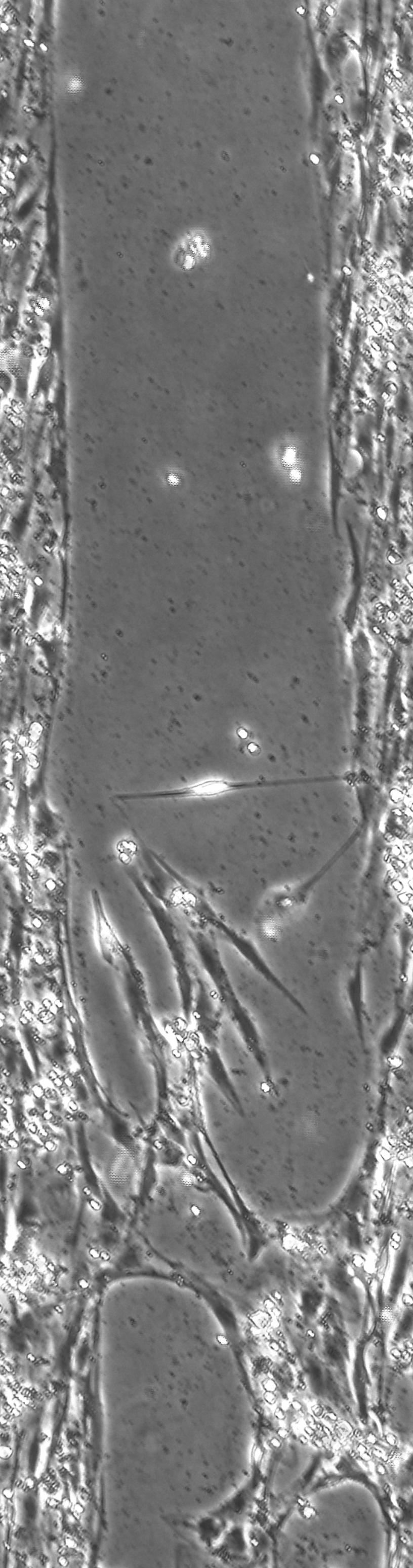

Supplement: S1 File — (ZIP) [file pone.0232518.s003.zip › 5 day/2.jpg]

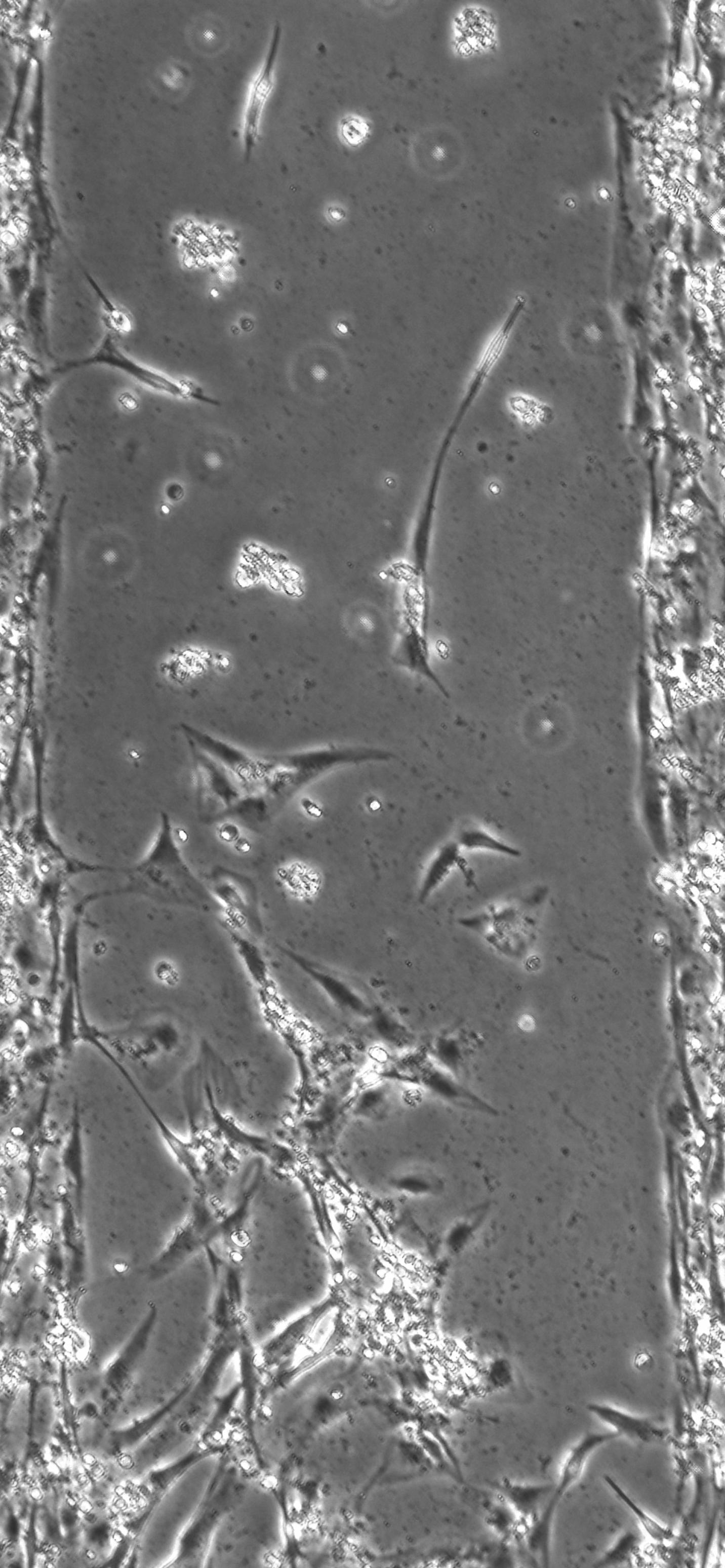

Supplement: S1 File — (ZIP) [file pone.0232518.s003.zip › 5 day/3.jpg]

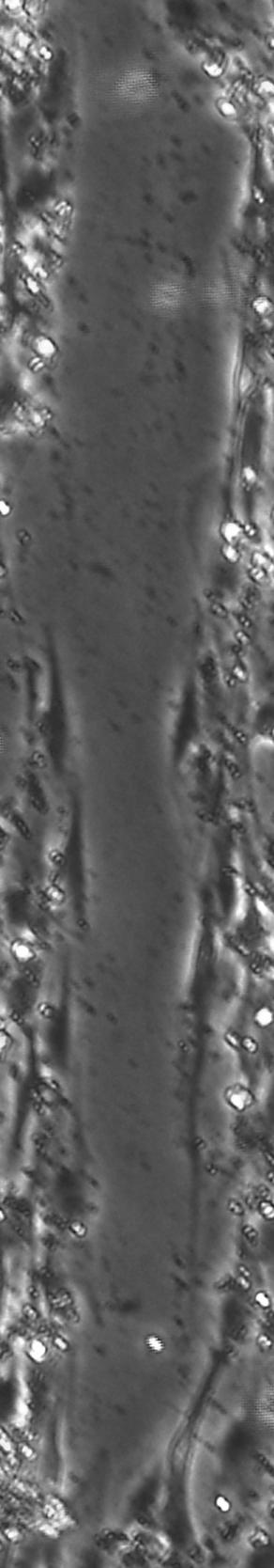

Supplement: S1 File — (ZIP) [file pone.0232518.s003.zip › 6 day/0.jpg]

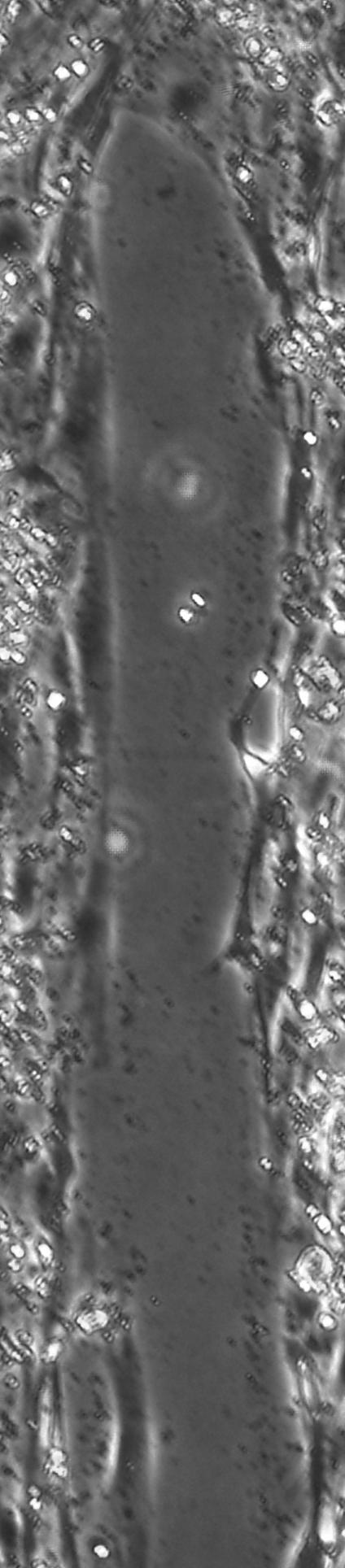

Supplement: S1 File — (ZIP) [file pone.0232518.s003.zip › 6 day/1.jpg]

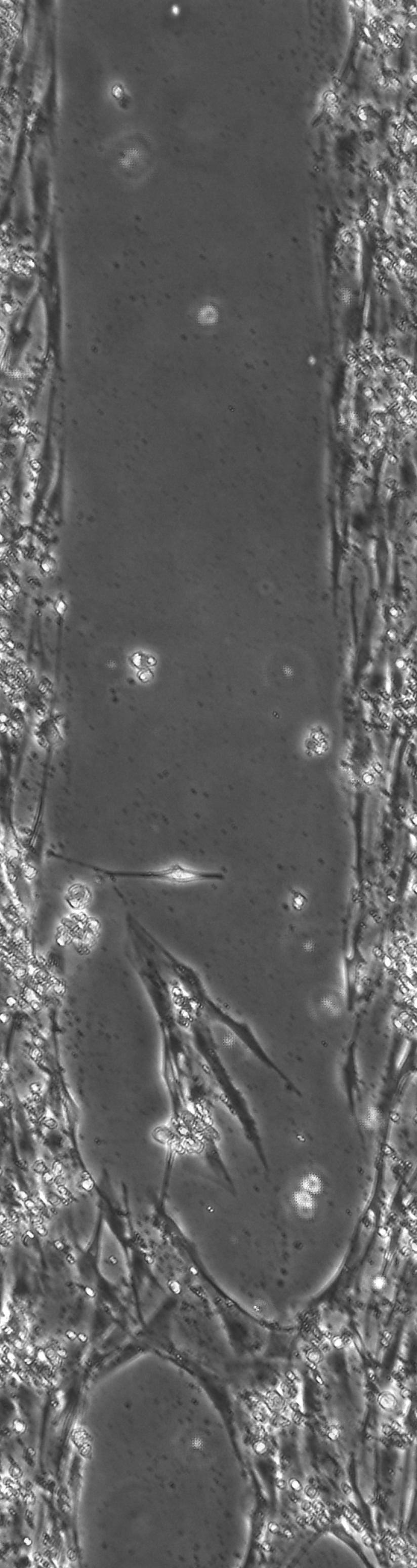

Supplement: S1 File — (ZIP) [file pone.0232518.s003.zip › 6 day/2.jpg]

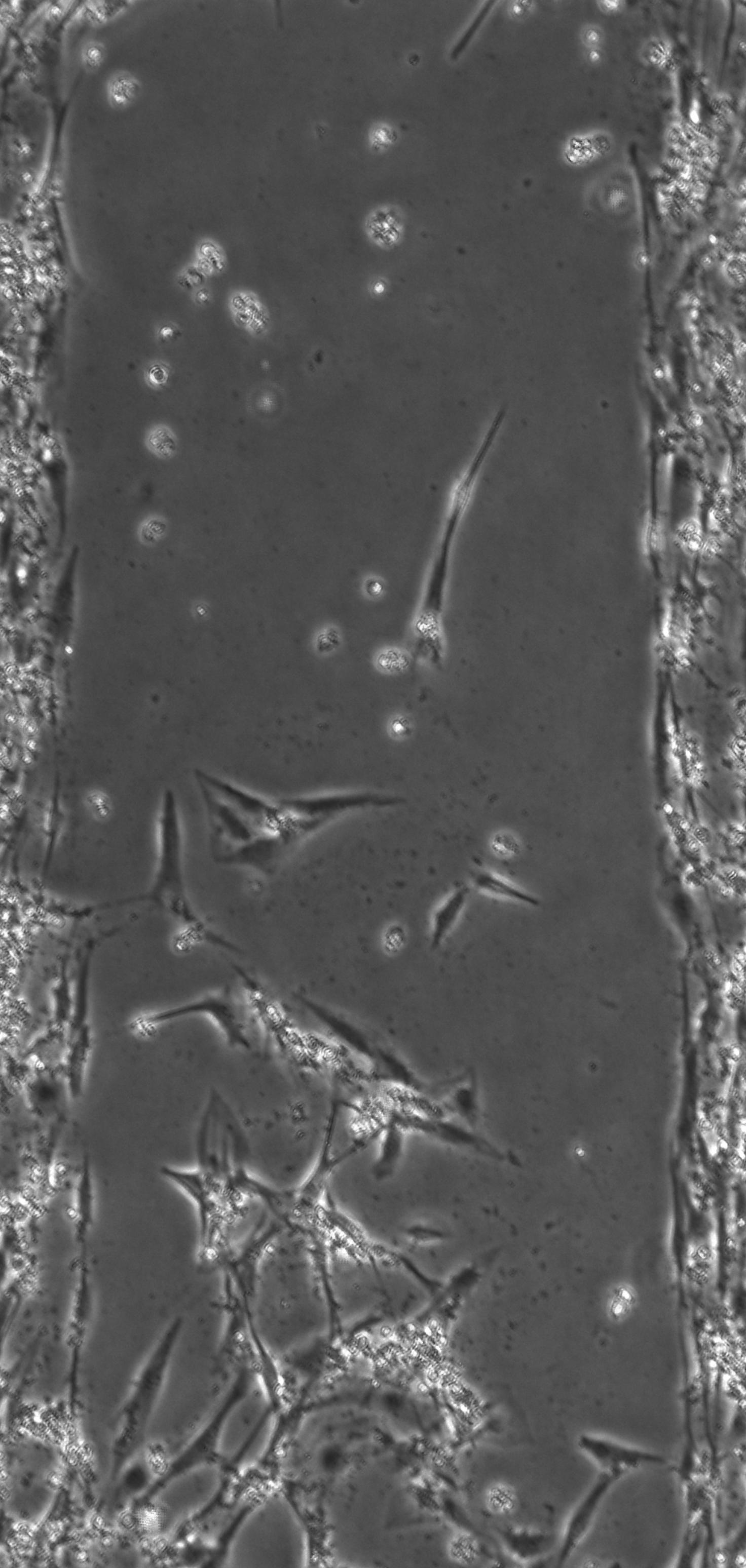

Supplement: S1 File — (ZIP) [file pone.0232518.s003.zip › 6 day/3.jpg]

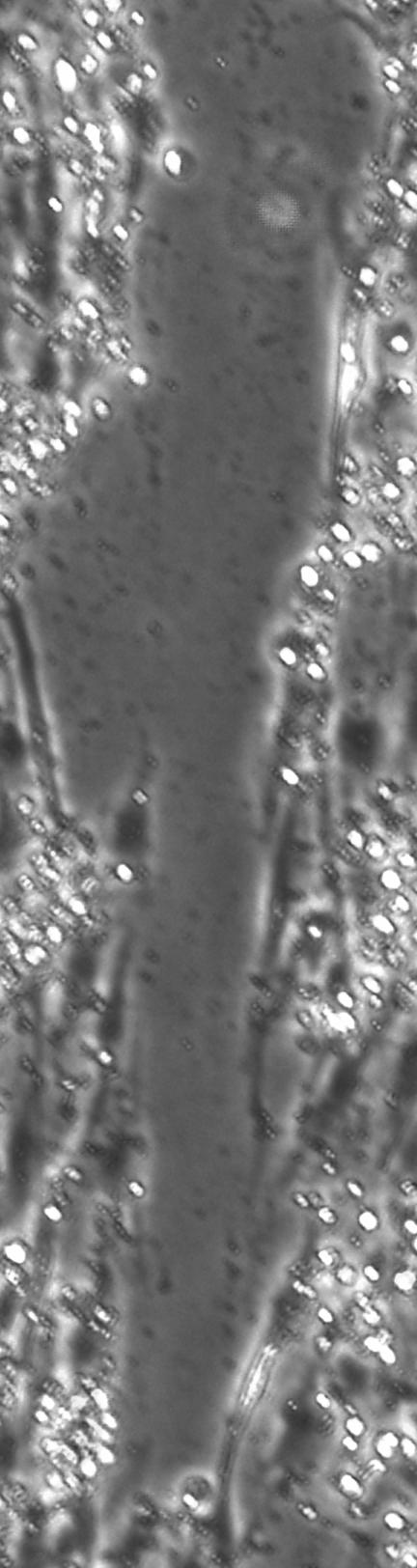

Supplement: S1 File — (ZIP) [file pone.0232518.s003.zip › 7 day/0.jpg]

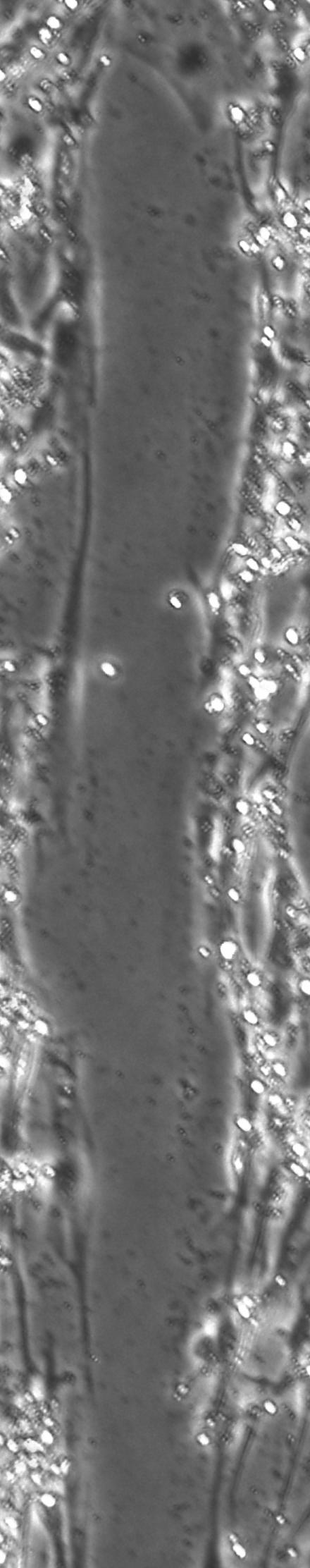

Supplement: S1 File — (ZIP) [file pone.0232518.s003.zip › 7 day/1.jpg]

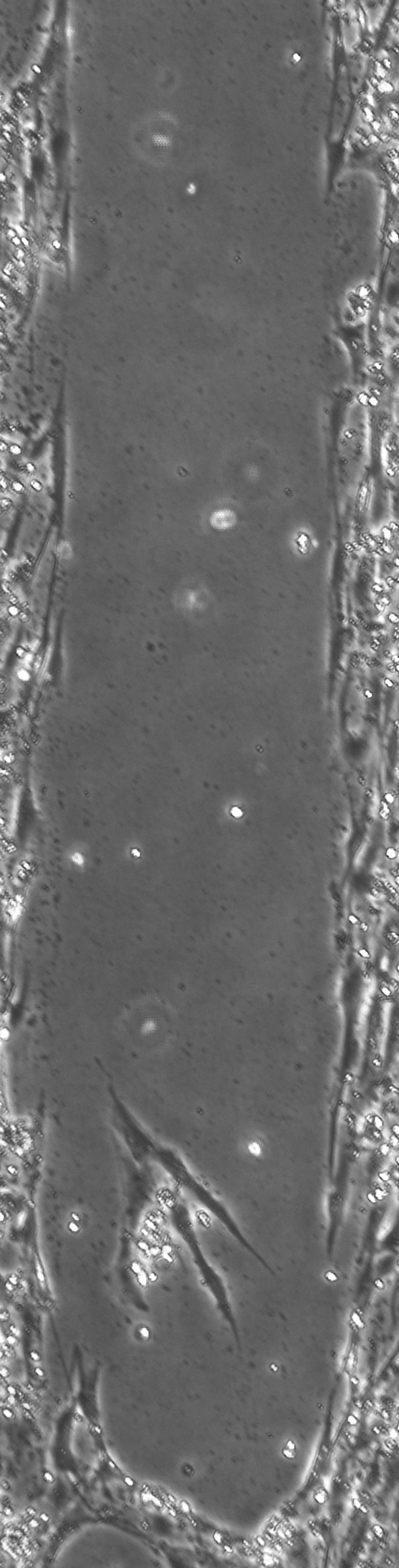

Supplement: S1 File — (ZIP) [file pone.0232518.s003.zip › 7 day/2.jpg]

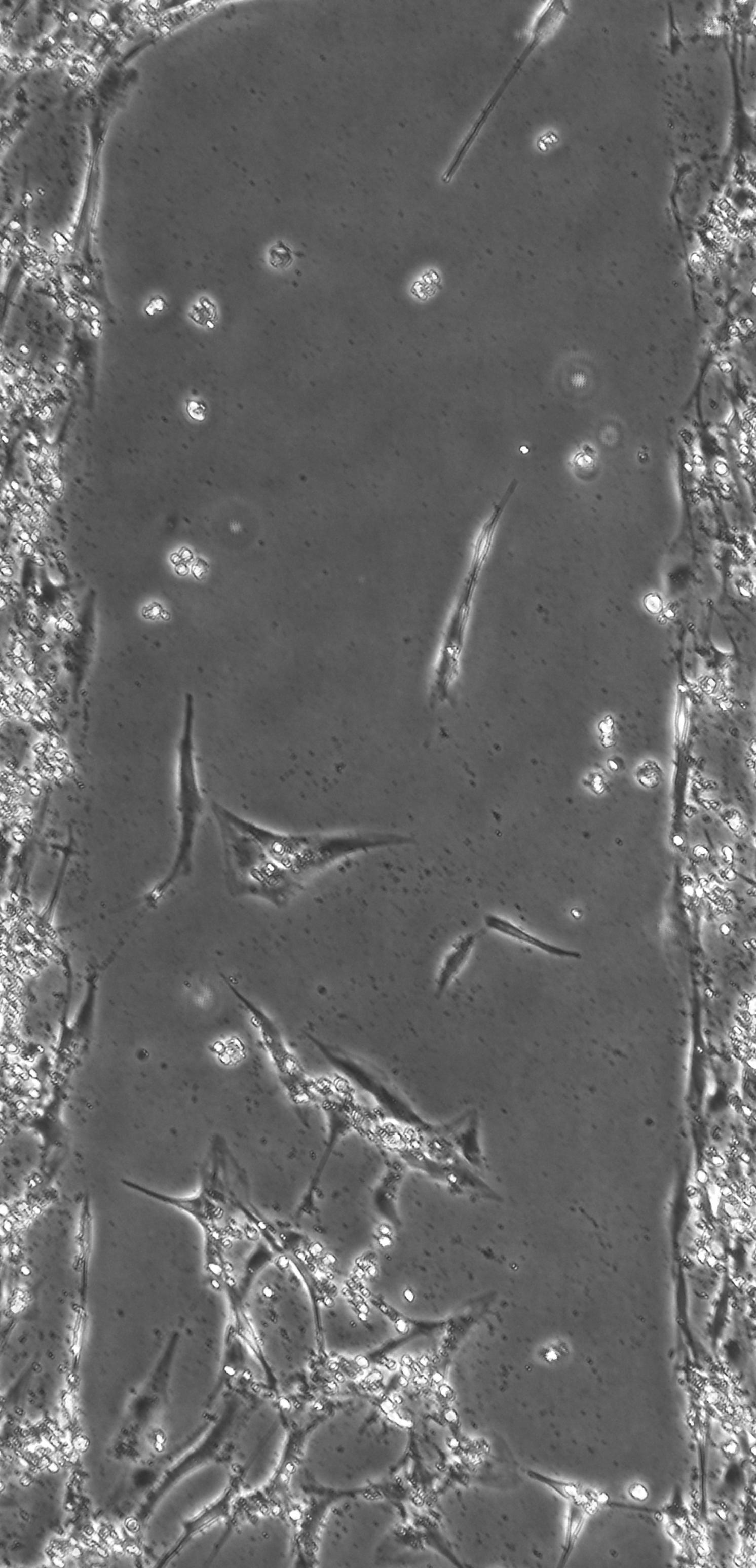

Supplement: S1 File — (ZIP) [file pone.0232518.s003.zip › 7 day/3.jpg]

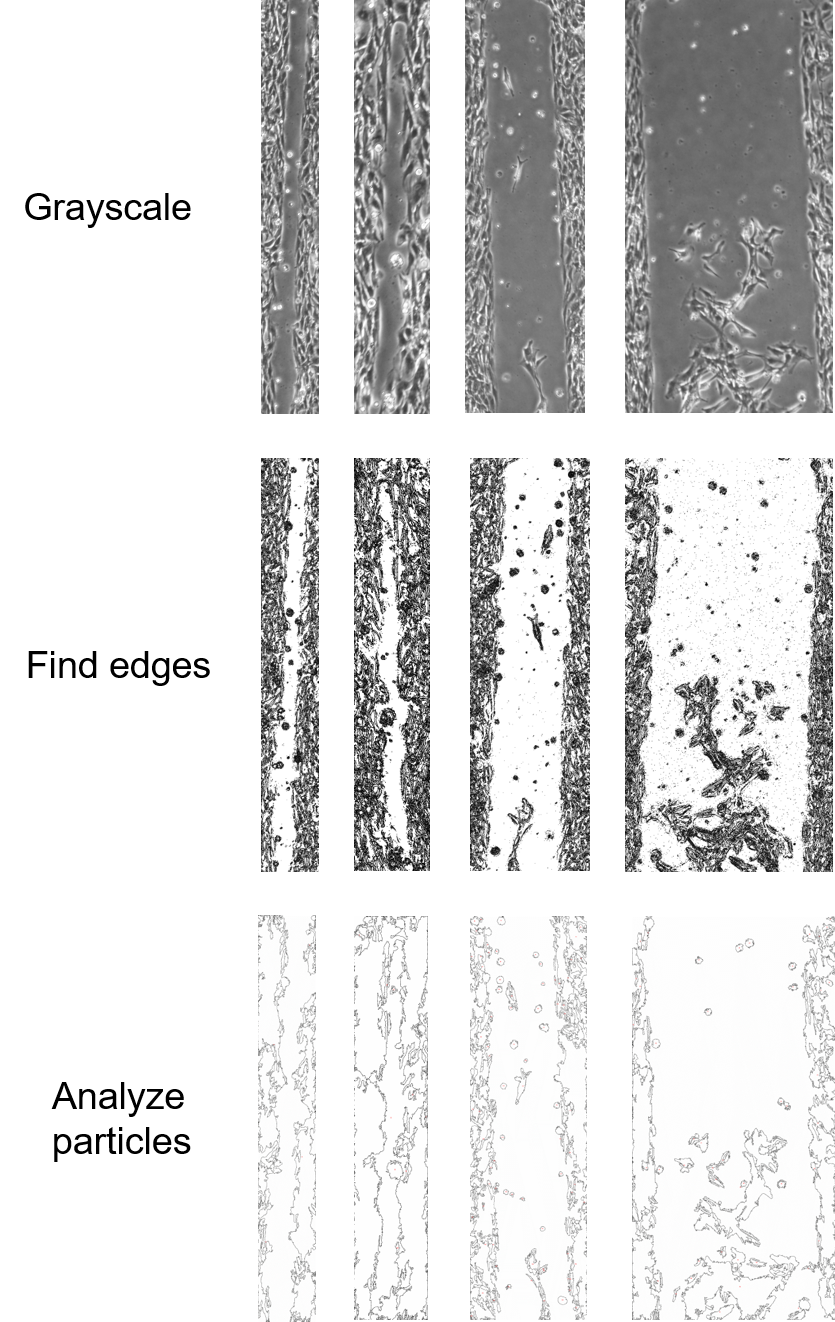


**Fig S1-1.** An example of the image process that converted the original image after the 1-d cell culture.

Supplement: S1 Fig — (DOCX) [file pone.0232518.s006.docx]
