# Supplementary material for: Flow analysis on microcasting with degassed polydimethylsiloxane micro-channels for cell patterning with cross-linked albumin
Source: PLoS One. 2020 May 20;15(5):e0232518. doi: 10.1371/journal.pone.0232518 (PMC7239381; doi:10.1371/journal.pone.0232518)
Supplement: S2 Fig — (DOCX) [file pone.0232518.s007.docx]

**
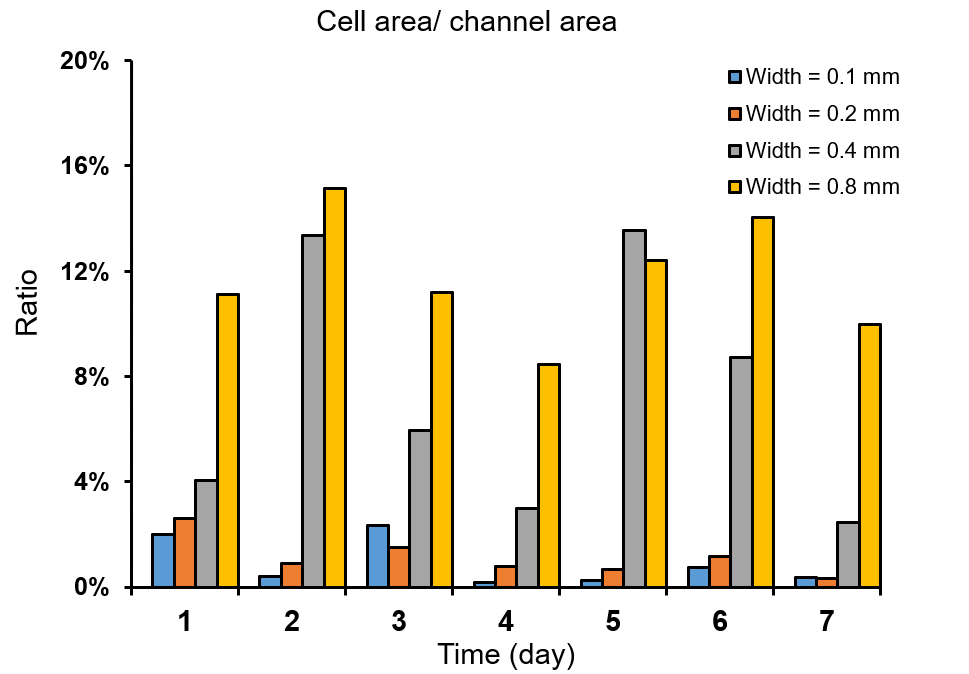
**

**Fig S1-2.** Area ratio of non-specific cell adhesion region and cell-blocking region in a one-week cell culture.
