# Supplementary material for: Flow analysis on microcasting with degassed polydimethylsiloxane micro-channels for cell patterning with cross-linked albumin
Source: PLoS One. 2020 May 20;15(5):e0232518. doi: 10.1371/journal.pone.0232518 (PMC7239381; doi:10.1371/journal.pone.0232518)
Supplement: S1 Table — (DOCX) [file pone.0232518.s008.docx]

**Table S2-1. Parameters of equations**

| Parameter | Meaning | Parameter | Meaning |
| --- | --- | --- | --- |
| F_air_ | Air flux | R_f_ | Flow rate resistance |
| C_1_ | Concentration of air in the atmosphere | η | Viscosity of liquid |
| C_0_ | Concentration of air inside PDMS after degassing | L | Length of micro-channel |
| D | Diffusivity of air in PDMS | W | Width of micro-channel |
| L_PDMS_ | PDMS thickness | H | Height of micro-channel |
| t_i_ | Post-vacuum idle time before loading | P_atm_ | Atmospheric pressure |
| P_c_ | Capillary pressure | K_1_, K_2_ | Empirical parameter |
