# Supplementary material for: Flow analysis on microcasting with degassed polydimethylsiloxane micro-channels for cell patterning with cross-linked albumin
Source: PLoS One. 2020 May 20;15(5):e0232518. doi: 10.1371/journal.pone.0232518 (PMC7239381; doi:10.1371/journal.pone.0232518)
Supplement: S2 Table — (DOCX) [file pone.0232518.s009.docx]

**Table S2-2. Value of parameters in the model.**

| Parameter | Value |
| --- | --- |
| η | -8.9 ╳ 10^-4^ Pa·s |
| D_PDMS_ | -3.4 ╳ 10^-9^ m^2^/s |
| P_atm_ | 10^5^ Pa |
| R | 8.31472 J/mol·k |
| T | 298 K |
