# Supplementary material for: Flow analysis on microcasting with degassed polydimethylsiloxane micro-channels for cell patterning with cross-linked albumin
Source: PLoS One. 2020 May 20;15(5):e0232518. doi: 10.1371/journal.pone.0232518 (PMC7239381; doi:10.1371/journal.pone.0232518)
Supplement: Attachment — Submitted filename: Response to Reviewers .docx [file pone.0232518.s010.docx]

**Response to Reviewers**

# Reviewer #1

**Overall Comment:**

Reviewer #1: With the pressure-driven pumping mechanism via micro channels provided by a degassed PDMS mold, the authors demonstrated, with rigorous analysis on experimental data and simulation results, a negative patterning methodology using cross-linked albumin that prevents cell adhesion. The authors also investigated the flow rate as functions of channel geometries and solution viscosity, as well as carried out simulations to better understand solution flow mechanisms. All experimental data and simulation results are made available via support information. The well-written manuscript provides a simple and useful approach for patterned cell cultivation by use of the pressure difference provided by degassed PDMS, which is expected to serve as a tool for researchers in biological science.

**Answer:**

Thank you very much for reviewing the manuscript carefully and giving many important comments. Based on the comments of all reviewers, we made extensive revisions as explained in the following answers. All text changes are shown in red color.

**Comment 1:**

The pumping mechanism is due to pressure differences generated by degassed PDMS, which relies on the material's porosity and air solubility. The authors perhaps should present a brief discussion on this aspect in their manuscript.

**Answer:**

Although the porosity and gas solubility of PDMS are important properties in the degassing and casting processes, our original discussion about material features was insufficient. PDMS has high gas permeability and the selectivity depends on the gas types; this is especially so for O_2_ and N_2_, major components of air, and it results in the high air solubility. Therefore, as long as the conditions are for a normal air atmosphere, this method works well for driving the casting solution. In our previous study [14], the gas that was dissolved in around 16% of the whole PDMS volume could be removed by degassing. And the accumulation of gas into the degassed PDMS after exposure to atmospheric pressure took around 10 min. In the case of higher porosity of PDMS, the gas-dissolving volume and accumulation recovery flux are assumed to increase with the pore size and distance between neighboring pores [27]. On the other hand, the high flux may cause a shortened operable time for driving the solution. Therefore, the optimal porosity should be considered from the viewpoint of practical use. We added the discussion in “Mechanism and simulation of degas-driven flow” of the section “Results and discussion”.

**After** **the revision:**

To estimate the flow rate of liquid in the micro-channel, this study employed a one-dimensional model (Fig. 3A) with the three physical conditions that influence: (1) the air solubility of PDMS, (2) the friction of liquid and (3) the capillary effect. As air diffuses into the PDMS, a pressure difference (vacuum pressure) is generated in the micro-channel relative to the atmospheric pressure. This vacuum pressure is the main driving force, which relates to features of PDMS, such as solubility, diffusivity and porosity. According to Henry’s law, the gas solubility in PDMS is described as:

$S=\frac{C}{p}$ (1)

where *S* [cm^3^ (standard conditions for temperature and pressure, STP)/(cm^3^ atm)] is the solubility coefficient and *C* [cm^3^ (STP)/ cm^3^] is the equilibrium gas concentration in PDMS at pressure *p* [atm]. It is known that the solubilities of O_2_ and N_2_ in PDMS are independent of the penetrant pressure but have a positive linear relationship with the temperature [26]. The ability for gas transfer depends on diffusivity in PDMS. Based on Fick’s law of diffusion, the air flux F at the surface of degassed PDMS can be described as:

$F_{air}\text{=}D\frac{\partial C}{\partial t}\left|\begin{matrix} \\ x=l \end{matrix}\right.\approx\frac{D_{0}(C_{1}-C_{0})}{l}\text{exp}\left(-t\frac{\pi^{2}D_{0}}{4l^{2}}\right)$ (2)

where *D* is the diffusion coefficient of gas in PDMS, *D_0_* is the average diffusion coefficient of gas in PDMS, *C_1_* is air concentration in PDMS before degassing, *C_0_* is the concentration of air inside PDMS after degassing, *C_0_* = *C* (*x*, 0) is the equilibrium concentration in the degassing step, and *l* is the thickness of the PDMS mold model. Therefore, high gas solubility and the actual gas diffusion material can lead to a high-pressure difference and larger air flux in the degassed PDMS. When it comes to the porosity, the gas-dissolving volume and accumulation recovery flux are assumed to be increased with the pore size and distance between neighboring pores [27]. On the other hand, the high flux may cause a shortened operable time for driving the solution. Therefore, the optimal porosity should be considered from the viewpoint of practical use.

**Comment 2:**

In section "Evaluation of viscosity effect for the flow rate", the authors assumed pressures of 30 to 40 kPa for their simulation. What are the grounds for assuming such high pressures? In fact, in Ref 14 the authors showed that the "remaining pressure" in degassed PDMS that was exposed to atmosphere pressure was less than 5 kPa within an hour. The authors perhaps want to reconsider their assumption on the pressure, or, at least, need to justify their assumption on the pressures they adopted in their simulation.

**Answer:**

Actually, we did not assume the pressure in the simulation, this pressure was calculated as a simulation result. As we hypothesized that the mechanism that drives degas-driven flow is the diffusion of air through the surface of the micro-channel into the PDMS by the accumulated vacuum pressure, we analyzed the pressure by simulating the process of diffusion of gas and fluid flow within the micro-channel. As for the remaining pressure, in Ref.14 we measured the vacuum pressure in PDMS elastomer, which showed the pressure changed in the sealed container with a volume of over 300 mL. Therefore, this result did not represent the pressure in the micro-channel having a 1 mL or smaller volume in the current study. Compared to production of a vacuum pressure in a larger container, it is easy to accumulate a vacuum in a micro-channel that has a much smaller volume relative to the container. From Fig. 4B in Ref.14 shown in the following figure, we can see that the pressure loss at a narrow micro-channel reached 20 kPa. In other words, the vacuum pressure must exceed the pressure loss to complete the casting process. We added this discussion to “Evaluation of viscosity effect for the flow rate” in the section “Results and discussion”.

**After: the revision**

From the simulation results, the vacuum pressure in the micro-channel could be estimated. The time-series of vacuum pressure was found to vary with the values of solution viscosity. And the maximum vacuum pressure was from 30 to 40 KPa at 40 to 60 s after solution application, which was over 10 times higher than the pressure loss at least (Fig 6B).

# Reviewer #2

**Overall Comment:**

This paper was focusing on Flow analysis on microcasting with degassed polydimethylsiloxane micro-channels. It is an quite interesting area about biomaterials. The author also used the quite hot topic of patterning. It would arouse of interests of audience.

**Answer:**

Thank you very much for the positive overall comment. Based on the comments of all reviewers, we made extensive revisions as explained in the following and all previous answers. All text changes are shown in red color.

**Comment 1:**

why the materials of agarose was used?

**Answer:**

In our previous work, we used agarose as the material for microcasting in the micro-channel. Agarose is known to be an extremely inert substance with respect to biomolecular adsorption, and it is widely available in biological laboratories. Furthermore, the prevention of biomolecular adsorption, especially for extracellular matrix molecules, enables the cell adhesion to be diminished, making a negative cell patterning approach possible. Based on these reasons, we used agarose again for the present study material in the previous study, though some difficulties such as the gelation step and long fabrication time remained.

**Comment 2:**

The influence of solution viscosity on the casting process should be further detailed.

**Answer:**

We recognized the influence of solution viscosity was different from that seen in a previous study using a PDMS micro-channel having an external degassed PDMS channel [34]. The main difference was the micro-channel structure; our study employed a dead-end micro-channel instead of the external degassed PDMS structure. In the dead-end structure, the exposed inner surface area of the PDMS micro-channel, producing the vacuum pressure, is decreased by flow of the casting solution, and conversely, the contact area is increased between the solution and PDMS wall, causing the viscous resistance. Reducing the exposed inner area causes bottleneck for obtaining the driving pressure, resulting in diminishing both flow rate and pressure loss. Therefore, we speculated decreasing the inner surface area influenced the flow rate of casting solution relatively more than the solution viscosity did. We added some explanation about the viscosity effect in “Evaluation of viscosity effect for the flow rate” of the section “Results and discussion.”

**After the revision:**

Therefore, the slight change of vacuum pressure was one of the reasons for occurrence of the viscosity-independent tendency. This tendency, which has not been reported in other related research studies [31-34], is mainly caused by the dead-end degassed PDMS channel. In this structure, the exposed inner surface area of the PDMS micro-channel, producing the vacuum pressure, is decreased by flow of the casting solution, and by contrast, the contact area is increased between the solution and PDMS wall, causing the viscous resistance. Reducing the exposed inner area causes a bottleneck for obtaining the driving pressure, resulting in diminishing both flow rate and pressure loss. Therefore, the decrease of the inner surface area was speculated to influence the flow rate of casting solution relatively more than the solution viscosity did.

**Comment 3:**

The introduction should add more information of the purpose.

**Answer:**

We added the purpose to the “Introduction” section.

**After the revision:**

As there is a difference in viscosity for agarose (34.6 mPa·s) and cross-linked albumin (1 mPa·s) at 30 °C, this study first investigated the influences of micro-channel dimensions and solution viscosity on the flow of solution in a micro-channel of a PDMS mold by both experiments and numerical simulation. Furthermore, to clarify the durability of cross-linked albumin under cell culture conditions, this study compared the cell patterns of mouse skeletal myoblasts C2C12 with microstructures made of native and cross-linked albumin.

**Comment 4:**

In Fig.5 d and h, the R value is very low, the author should give more explanations

**Answer:**

Actually, the low R value was caused by the viscosity-independent tendency, and we discussed this reason in the section “Evaluation of viscosity effect for the flow rate”. We added the explanation of the low R value in the revised version.

**After the revision:**

However, the low coefficient of determination R^2^ (0.0074 for experimental data and 0.0087 for simulation data) indicated that there was no correspondence between viscosity and flow rate. In other words, this liquid with a wide range of viscosity from 1 to 575 mPa·s could be driven into the micro-channel with little change of flow rate. As the pressure difference *∆P* dominated the flow rate, it was assumed that the small variation of pressure at different viscosities caused the viscosity-independent tendency. This is described in more detail in the section “Evaluation of viscosity effect for the flow rate”.

# Reviewer #3

**Overall Comment:**

The manuscript applied degassed PDMS’s vacuum pressure property for fabrication of patterned cross-linked albumin, which demonstrate the control on the cell culturing alignment. The results are fairly good for being considered by this journal.

**Answer:**

Thank you very much for the positive overall comment. Based on the comments of all reviewers, we made extensive revisions as explained in the following and previous answers. All text changes are shown in red color.

**Comment 1:**

English proofreading is mandatorily needed:

Line 3, Page 3 – “… has …” should be “… have …”

**Answer:**

Based on the comment, the manuscript has been carefully reviewed by a native English speaker who specializes in editing scientific papers.

**After the revision:**

"In the field of cell biology, because the spatial arrangement of cells is an important factor in morphogenesis and tissue functions [1,2], artificially generated spatial patterns of cells have been often used."

**Comment 2:**

Line 8, Page 3 – what is exactly “surrounding environments”?

**Answer:**

From this comment we realized our explanation was unclear and we rewrote it. Here, the “surrounding environments” was meant to be a stripe micropattern of polymer brushes and we said that in the revised sentence.

**After the revision:**

In tissue engineering, myocytes well aligned by a stripe micropattern of polymer brushes have a potential to produce higher contraction than that in the native condition [6,7].

**Comment 3:**

Line 10 – Line 12, Page 3 – this micro contact printing technique should be briefly discussed for its role to pattern the cell adhesive substance (through ink transferring).

**Answer:**

Based on the comment, we added a brief discussion about the micro contact printing technique.

**After the revision:**

As a cell spatial arrangement technique, the straightforward and inexpensive micro contact printing method has been used, in which a cell adhesive substance such as fibronectin is transferred to a cell culture surface as an ink [9,10]. Although micro contact printing is simple and easy to use, the pattern resolution is limited by the stamp deformation and ink mobility.

**Comment 4:**

Line 14, Page 3 – why did the authors use “on the other hand”?

**Answer:**

From the reviewer’s comment we realized our description was not suitable and we deleted it.

**Comment 5:**

Line 16 – Line 18, Page 3 – “In the … into the mold” should be whole rewritten.

**Answer:**

Our description was not concise and we rewrote it.

**After the revision:**

The authors have succeeded in patterning cells using a microstructure cast of agarose, one of the non-cell-adhesive substances [14,15]. In these two studies, a casting solution was driven into a degassed polydimethylsiloxane (PDMS) mold by the accumulated vacuum pressure in the PDMS.

**Comment 6:**

Line 4, Page 4 – “… the viscosity large …” should be rewritten.

**Answer:**

We rewrote the sentence.

**After the revision:**

Because this vacuum pressure is caused by the diffusion of air into spaces inside PDMS elastomer [16,17], the time in which the vacuum pressure is produced is limited with a time constant of around 10 min [14]. It is known that when a high viscosity solution flows in a narrow channel, it generates a large pressure loss, reducing the flowability. This might cause the introduction of solution to take longer than expected from vacuum production time constant, resulting in insufficient casting of the microstructure.

**Comment 7:**

Line 13, Page 18 – should be “slightly”

**Answer:**

We changed the word.

**After the revision:**

The difference in width between the cross-linked albumin pattern and the real cell pattern decreased and then slightly increased (Fig 7H-K).

**Comment 8:**

What is “analogous gas – based cell repellent surface patterning method”? The manuscript should briefly mention about in the introduction.

**Answer:**

Our explanation was lacking and we added details.

**After the revision:**

The authors have also developed a gas-based cell repellent surface patterning method by using the degassed PDMS to suck a special gas into the micro-channel for surface treatment [18,19].

**Comment 9:**

Line 13, Page 4: “To cope with the viscosity issue …” is quite unsmooth from the aforementioned background. I did not see any viscosity impacts before.

**Answer:**

We changed the content in the revised vision.

**After the revision:**

To reduce the complexity of using the agarose casting and add operability of the cell patterning, a low viscosity aqueous solution of cross-linked albumin was introduced as a casting solution and it formed the pattern for cell culture (Fig 1A-G). In recent years, simple cell patterning has been realized by cross-linking albumin, which is a protein that is abundant in serum, and preparing functional materials that can repel cell adhesion [23,24]. Because the cross-linked albumin solution has a one-way dehydration process from solution to solid and an insoluble property after dehydration, the gelation step required with an agarose solution can be eliminated. As there is a difference in viscosity between agarose (34.6 mPa·s) and cross-linked albumin (1 mPa·s) at 30 °C, this study first investigated the influences of micro-channel dimensions and solution viscosity on the flow of solution in a micro-channel of a PDMS mold by both experiments and numerical simulation. Furthermore, to clarify the durability of cross-linked albumin under cell culture conditions, this study compared the cell patterns of mouse skeletal myoblasts C2C12 with microstructures made of native and cross-linked albumin. Finally, this study compared the cell patterns of mouse skeletal myoblasts C2C12 with microstructures made of native and cross-linked albumin.

**Comment 10:**

The quality of figures should be improved with higher resolution.

**Answer:**

We had updated the revised version with high-resolution figures.

**Comment 11:**

I did not quite understand the description in Line 2, Page 6: “diffusion of air through the walls …” there is vacuum pressure in the microchannel. Why did the author say “diffusion of air … into the bulk PDMS”.

**Answer:**

Our description was insufficient and inaccurate, which caused misunderstanding. The PDMS has a gas solubility and permeability. Therefore, the degassed PDMS elastomer can dissolve ambient gas molecules through the surface. In the case of a closed space such as a micro-channel, this gas absorption provides the vacuum pressure. In our casting approach, we employed this vacuum pressure generated by the degassed PDMS elastomer as a driving force to suck the casting solution into dead-end microchannel structures in a PDMS mold. Based on the comment, we added details about this pumping mechanism in the revised version. We also added several sentences about the PDMS properties such as solubility, diffusivity, and porosity in “Mechanism and simulation of degas-driven flow” of the section “Results and discussion”.

**After the** **revision:**

As air diffuses into the PDMS, a pressure difference (vacuum pressure) is generated in the micro-channel relative to the atmospheric pressure. This vacuum pressure is the main driving force, which relates to features of PDMS, like solubility, diffusivity and porosity. According to Henry’s law, the gas solubility in PDMS is described as:

$S=\frac{C}{p}$ (1)

where *S* [cm^3^ (standard conditions for temperature and pressure, STP)/(cm^3^ atm)] is the solubility coefficient, and *C* [cm^3^ (STP)/ cm^3^] is the equilibrium gas concentration in PDMS at pressure p [atm]. It is known that the solubilities of O_2_ and N_2_ in PDMS are independent of the penetrant pressure but have a positive linear relationship with the temperature [26]. The ability for gas transfer depends on diffusivity in PDMS. Based on Fick’s law of diffusion, the air flux F at the surface of degassed PDMS can be described as:

$F_{air}\text{=}D\frac{\partial C}{\partial t}\left|\begin{matrix} \\ x=l \end{matrix}\right.\approx\frac{D_{0}(C_{1}-C_{0})}{l}\text{exp}\left(-t\frac{\pi^{2}D_{0}}{4l^{2}}\right)$ (2)

where *D* is the diffusion coefficient of gas in PDMS, *D_0_* is the average diffusion coefficient of gas in PDMS, *C_1_* is air concentration in PDMS before degassing, *C_0_* is the concentration of air inside PDMS after degassing, *C_0_* = *C* (*x*, 0) is the equilibrium concentration in the degassing step, and *l* is the thickness of the PDMS mold model. Therefore, high gas solubility and the actual gas diffusion material can lead to a high pressure difference and larger air flux in the degassed PDMS. When it comes to the porosity, the gas-dissolving volume and accumulation recovery flux are assumed to be increased with the pore size and distance between neighboring pores [27]. On the other hand, the high flux may cause a shortened operable time for driving the solution. Therefore, the optimal porosity should be considered from the viewpoint of practical use.

This study hypothesized that the principle mechanism of driving liquid is that of air diffusion through the micro-channel surface into the PDMS mold and pressure-driven Hagen–Poiseuille-like ﬂuid ﬂow within micro-channels, and that the fluid will replace the decreasing air flux in time. Hence, the volumetric flow rate *Q* can be described by the following equation [28,29]

$Q\propto F_{air}\text{=}k\frac{A(t)D_{0}(C_{1}-C_{0})}{l}\text{exp}\left(-t\frac{\pi^{2}D_{0}}{4l^{2}}\right)$ (3)

$Q=\frac{\Delta P}{R_{f}}$ (4)

where *F_air_* is the air flux, *k* is an empirical factor related to the wetting property and capillary effect of solution. *A(t)* is the surface that air diffuses through. R_f_ is the flow resistance caused by the friction of liquid and *∆P* is the pressure difference between pressure in a micro-channel (*P_i_*) and atmospheric pressure (*P_atm_*).

$\Delta P=P_{atm}-P_{m}+P_{c}$ (5)

*P_c_* is the capillary pressure caused on the interface of the solution in a rectangular cross-sectional micro-channel and it is given by the following equation [30]

(6)

where *θ_c_* is the contact angle (CA) of the solution with the micro-channel wall; *θ_b_* is the CA of the solution with the micro-channel bottom (Fig 3B); *γ* is surface tension of the solution; and *h* and *w* are height and width of the micro-channel,respectively.
